# Foreword to the special virtual issue on *X-ray spectroscopy to understand functional materials: instrumentation, applications, data analysis*

**DOI:** 10.1107/S1600577524008695

**Published:** 2024-10-21

**Authors:** Elisa Borfecchia, Kirill A. Lomachenko, Kristina Kvashnina

**Affiliations:** a University of Turin 10125Turin Italy; b ESRF – The European Synchrotron, 38043Grenoble Cedex 9, France

**Keywords:** X-ray absorption spectroscopy, functional materials, synchrotron radiation

## Abstract

Foreword to the virtual focused issue of *Journal of Synchrotron Radiation* on *X-ray spectroscopy to understand functional materials: instrumentation, applications, data analysis*.

Synchrotron-based X-ray spectroscopy currently represents an essential tool to disclose the structural and physico-chemical properties of advanced materials and to track their response relevant to target functional applications. Today, impressive developments at dedicated beamlines guarantee unprecedent resolution in the time, space and energy domains, eventually under operando conditions and with quasi-simultaneous combination of different techniques. The inherent elemental selectivity and chemical sensitivity of X-ray spectroscopy have been substantially augmented by emerging data analysis methods based on statistical and machine learning (ML) approaches.

The present virtual focused issue (https://journals.iucr.org/special_issues/2024/xrayspectroscopy) collects exciting examples on the key role of X-ray absorption spectroscopy (XAS) to understand functional materials, emphasizing recent advances in methods, applications and data analysis by leading teams in the field, from both synchrotron facilities and academic groups. The focused collection encompasses high-impact research fields across physical and chemical science, from (photo/electro-)catalysis, to energy storage and conversion, passing through fundamental problems in coordination chemistry and materials science.

The focused collection is dedicated to Professor Carlo Lamberti, a brilliant, all-round X-ray scientist, who unexpectedly passed away in February 2019 at the age of 54.

In several cases, the contributions present innovative data collection and pre-treatment methods, aimed at pushing the limits of X-ray spectroscopy and expanding the accessible information for the user community. In this context, Frenkel and co-workers report on a novel approach to automate the removal of Bragg peaks from the XAS spectra of crystalline samples (Shimogawa *et al.*, 2024); Glatzel and co-workers discuss the issue of properly normalizing high-energy-resolution fluorescence-detected (HERFD)-XANES spectra, collected on spectrometer-based beamlines such as ID26 at the European Synchrotron Radiation Facility (ESRF) (Bugarin *et al.*, 2024); Briois and co-workers provide an in-depth review on the potential of hyperspectral full-field quick-EXAFS imaging implemented on the ROCK beamline at Synchrotron Soleil with an emphasis on applications to functional materials monitored under process conditions (Briois *et al.*, 2024). The importance of designing innovative cells, essential in the field of operando studies, is also underlined. In this respect, Bugaev and co-workers describe a custom-made *operando* photocatalytic cell employed to effectively monitor by XAS the formation steps of a Pd/TiO_2_ photocatalyst (Kozyr *et al.*, 2024).

The focused collection also includes several reports on innovative data analysis approaches, employed to interrogate X-ray absorption spectra in both the XANES and EXAFS region, and successfully applied to ground-breaking research in chemistry and materials science. In particular, Soldatov and co-workers illustrate how XANES fitting could allow enhanced structural sensitivity to first- and second-shell interatomic distances, especially valuable in cases where EXAFS is not easily accessible (Abrosimov *et al.*, 2024). Martini and co-workers present a timely case study on the use of ML-assisted XANES analysis applied to *operando* data obtained during electrocatalytic CO_2_ reduction over Co single-atom catalysts (Martini *et al.*, 2024). On the same line, Grunwaldt and co-workers report on the implementation of *RefXAS*, an open-access database of XAS spectra, responding to the urgent need for training datasets conceived according to the FAIR principles, to fully unleash the potential of ML-based methods in the field of X-ray science (Paripsa *et al.*, 2024). Contributions by Boscherini and co-workers and Glatzel and co-workers illustrate the potential of HERFD-XANES, as an unprecedented tool to reveal subtle spectral variations and an essential experimental benchmark for *ab initio* simulations (Piccioni *et al.*, 2024; Suarez Orduz *et al.*, 2024). Finally, d’Acapito and co-workers address the use of *ab initio* molecular dynamics in simulating EXAFS spectra, taking layered materials as an example (d’Acapito *et al.*, 2024).

With these reports, we aim at reflecting the brilliant and multi-faceted contribution provided by Professor Lamberti in the field of synchrotron-based characterization of functional materials, tirelessly building bridges between research fields and communities, while making countless friends!

Carlo Lamberti, born in 1964, obtained his MSc degree in Physics in 1988, *cum laude*. In the 1988–1993 period, he worked in the CSELT laboratories in Torino (Italy) on the characterization of the interfaces of III–V semiconductor heterostructures with 4 K photoluminescence, high-resolution XRD and X-ray absorption spectroscopies. He presented his PhD defence in solid state physics on this topic in Rome in September 1993. ▸

In that period, Carlo’s research interests started to shift towards physical chemistry and materials science, during a post-doctoral position at the Department of Inorganic, Physical and Materials Chemistry of Torino University, within the group of Professor Adriano Zecchina. In the same department, he became Faculty Researcher in 1998 and then Associate Professor in 2006. Starting from the characterization of III–V semiconductor heterostructures, Carlo’s research encompasses an impressive number of systems and problems: interaction of small molecules at the surfaces of oxides, zeolites and metal-organic frameworks; porous materials for molecular storage; catalyst characterization under *in situ*/*operando* conditions with vibrational, electronic and X-ray techniques; and non-stoichiometric oxides explored combining neutron and photon probes, by both diffraction and spectroscopy.

Over two decades, Carlo become an internationally recognized expert of synchrotron radiation in the characterization of materials, performing more than 200 experiments at most of the European synchrotrons (and beyond). He also served for several years as member and chair of the proposal review committees at the ESRF and the Swiss Light Source.

Carlo was also extremely active and committed in disseminating the results of this research: he (co)-authored more than 300 research papers that appeared in the most important journals in the field of physics, chemistry and material science, including authoritative reviews in *Chemical Reviews*, *Chemical Society Reviews* and *Reviews of Modern Physics*. Now, in August 2024, according to Google Scholar, his *h*-index has reached 108, and his works have received more than 45000 citations to date. He has also presented, personally or via colleagues, more than 500 congress contributions, among them more than 20 invited lectures in Europe, India, Canada, Japan and USA, always ‘activating’ the audience with smart questions and constructive comments. Beyond bibliometrics, these numbers give us an idea of the impact that Carlo’s research had in the relevant scientific communities – and he would have been very proud of that.

His collaboration network worldwide was authentically impressive, including both academic and industrial partners, such as AVAGO, ENI, SüdChemie, EVC, INEOS, Chimet, BASF and Haldor Topsøe A/S. Noteworthy, together with Professor Alexander Soldatov, he has been the Principal Investigator of a mega-grant of the Russian Federation Government at the Southern Federal University in Rostov-on-Don in the 2014–2018 period, and Scientific Director of the Smart Materials International Research Institute at the same university. Carlo put all his enthusiasm and commitment in rich scientific exchanges with the Rostov-on-Don group, yielding outstanding results in terms of excellent publications and training of numerous young researchers. In 2018 Carlo Lamberti was awarded the degree of Doctor Honoris Causa by the Southern Federal University in recognition of his remarkable achievements.

Carlo was also a versatile and passionate teacher, easily ranging from quantum physics to materials chemistry. He was among the founders of the MaMaSELF Erasmus Mundus Joint Master in 2007 (https://www.mamaself.eu/), and passionately contributed to this unique program for more than a decade. He also supervised a number of Master’s and PhD thesis projects, inspiring so many of his former students in pursuing their career as beamline scientists at large-scale facilities as well as academic/industrial researchers being frequent users of synchrotron sources worldwide.

Carlo left us impressive examples of how X-ray spectroscopy can disclose the intimate nature and reactivity of functional materials. His impressive scientific production testifies his broad interests and exceptional capacity in building bridges among user communities working with synchrotron radiation. This focused collection also represents a tribute to Carlo’s contagious enthusiasm for novel developments and exciting challenges related to X-ray spectroscopy in materials science.

## Figures and Tables

**Figure 1:**
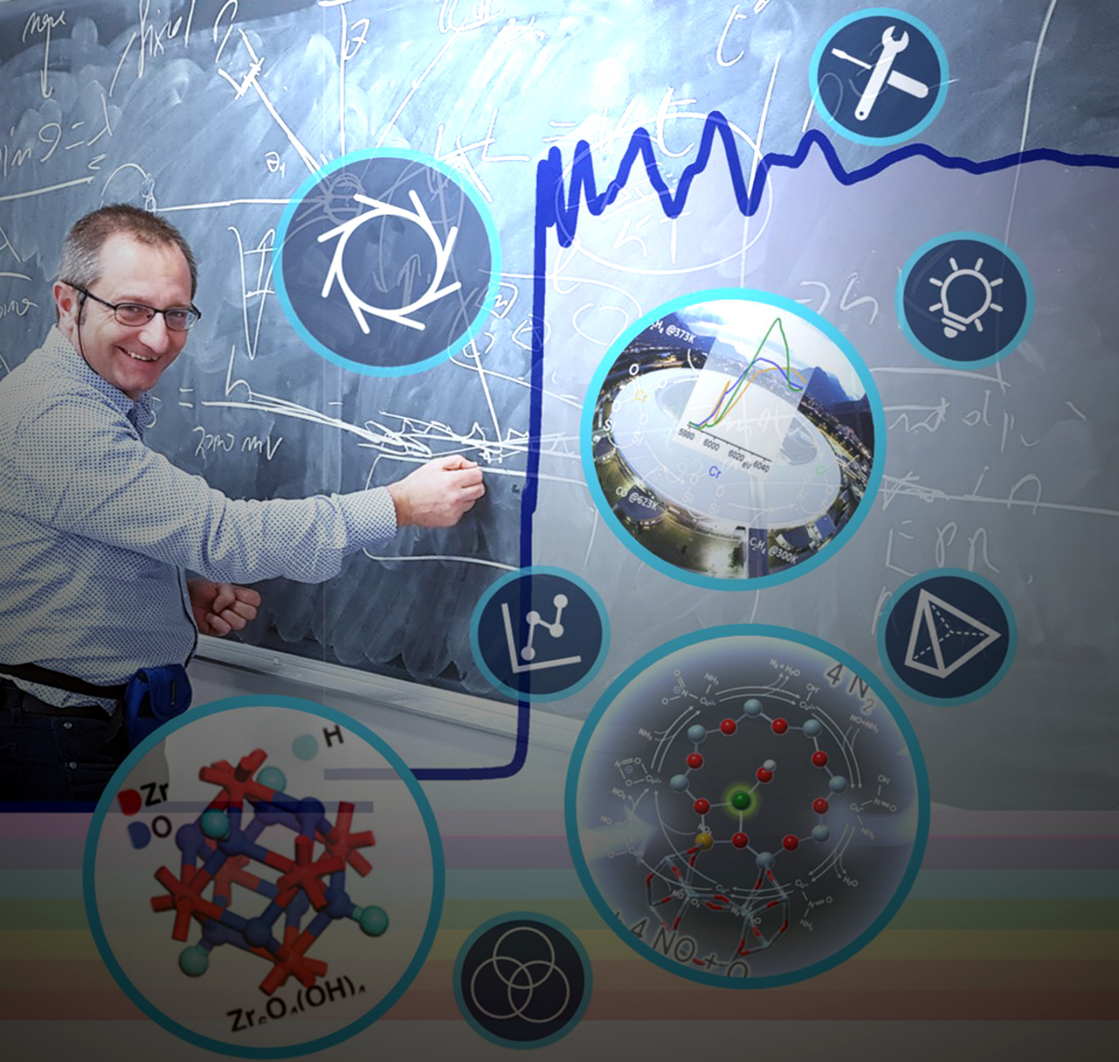
Carlo Lamberti, to whom this special virtual issue is dedicated.
